# Epidemiological Variations in the Global Burden of Psoriasis, an Analysis With Trends From 1990 to 2017

**DOI:** 10.3389/fmed.2021.585634

**Published:** 2021-02-02

**Authors:** Chen Peng, Xin Xu, Wenjuan Chen, Xingzi Li, Xuemei Yi, Yangfeng Ding, Ning Yu, Jiajing Lu

**Affiliations:** ^1^Department of Dermatology, Shanghai Skin Disease Hospital, Tongji University School of Medicine, Shanghai, China; ^2^Institute of Psoriasis, Tongji University School of Medicine, Shanghai, China

**Keywords:** psoriasis, global burden, gender inequality, geographic and socioeconomic disparity, epidemiology

## Abstract

**Background:** Although there have been many epidemiological studies, research focusing on psoriasis' health burden on a global scale is still lacking. Trends and variations in the global health burden of psoriasis are evaluated by time, age, gender, geographical location, and socioeconomic status, using disability-adjusted life years (DALYs) from the Global Burden of Disease Study.

**Methods:** The health burden of psoriasis was evaluated by DALYs, which combined years lost to disability (a morbidity component) with years of life lost (a mortality component). The global and national DALYs number, crude DALYs rate, and age-standardized DALYs rate were obtained from the GBD 2017 study database. The corresponding human development index (HDI) was collected from the United Nations Development Programme.

**Results:** From 1990 to 2017, the DALYs number and crude DALYs rate due to psoriasis increased by 73 and 22%, respectively. In comparison, the age-standardized DALYs rate showed a slight increase. Patients in the age range of 65–69 years bear a more significant psoriasis burden. Both males and females showed an increasing trend in burden caused by psoriasis over the past 27 years, with females bearing a more significant psoriasis burden than males. The health burden of psoriasis was substantially unequal in geography with a Gini coefficient of 0.27. The concentration indexes indicated a socioeconomic associated inequality in psoriasis burden with values of 0.22, accounting for 48.64% variance across countries (R2 = 0.4864, *p* < 0.001). Between-nation inequality in the distribution of psoriasis burden continued to decline throughout the past 27 years. Gini coefficients of psoriasis burden decreased from 0.280 in 1990 to 0.265 in 2017. The concentration indexes indicated the same trend with 0.236 in the 1990s and 0.223 in 2017.

**Conclusions:** Global health progress in psoriasis together with inequality in the past few decades. Although the inequality of psoriasis burden has shown some improvement during the past 27 years, disparities still exist in age, gender, geographical location, as well as socioeconomic status. The findings of this study highlight the global importance of psoriasis and is important in policy planning for psoriasis services on a global scale.

## Introduction

Psoriasis is a chronic, immune-mediated inflammatory disorder frequently characterized by relapsing thick scaling plaques, with or without nail and joint involvement. While psoriasis is usually not life-threatening, patients can incur physical discomfort (e.g., pruritus, pustule, arthralgias, etc.) as well as psychosocial harm (1) which prevent sufferers from achieving their full-life potential. Psoriasis patients always suffer lifelong impacts because of the chronic nature of the condition (2). Besides psoriasis-related discomfort or disability, patients have to bear the direct, indirect, and intangible economic burden (2) as well as a loss of work productivity due to the disease (3, 4). Moreover, psoriasis patients often have to face stigma in social settings. Although there have been many epidemiological studies, research focusing on the health burden of psoriasis, on a global scale, is still lacking.

The health burden of a disease refers to the total loss of patients, their families, and society caused by disease, disability and/or pre-mature death, including life loss, economic loss, and the deterioration of life quality (5). Disease burden is often measured by a prevalence rate (i.e., the count of existing cases in a given period of time and place) or by an incidence rate (i.e., the count of new cases over a period of time or person-years) which are unilateral for the evaluation of health burden. Different to other indicators, in the Global Burden of Diseases Study (GBD study), the health burden of psoriasis is evaluated by disability-adjusted life years (DALYs) (6) which combines years lost to disability (a morbidity component) with years of life lost (a mortality component) (7). DALYs consider the length of life and the quality of life and is already widely used in many countries as a basis to formulate health policies in global disease evaluation and measurement.

Our study used the most recent DALYs data available from the GBD 2017 study to explore the trends and variations in health burden of psoriasis from 1990 to 2017 globally, providing information for health policy planning and resource allocation.

## Materials and Methods

### Study Design

This study is an international, comparative burden-of-disease study.

### Global Burden of Psoriasis

The GBD 2017 study provides particular perspectives for the health burden of over 350 diseases and injuries across 196 countries/territories, supporting comparisons of the magnitude of diseases, injuries, and risk factors across age groups, sexes, countries, regions, and time from 1990 to the present day. The Institute for Health Metrics and Evaluation (IHME), an independent global health research center at the University of Washington, led the GBD 2017 study. Population figures are estimated based on World Population Prospects: 2015 Revision, from the United Nations Population Division, and the WHO Human Mortality Database. Country start and end dates, notes, and sovereignty comes from the CIA World Factbook. Organization start and end dates and acronyms generally come from the organization's website, as do the city and country of their location. The global burden of psoriasis was estimated by the GBD 2017 study in terms of DALYs number, crude DALYs rate, and age-standardized DALYs rate globally. The algorithm for calculating DALYs was detailed in the GBD 2017 study methodological reports (8). Crude DALYs rate was calculated by accounting for population size, and the age-standardized DALYs rate took the age-structure and population size into consideration. We obtained the data from the GBD study database (http://ghdx.healthdata.org/gbd-results-tool) (Global Burden of Disease Study 2017). We collected the following data concerning psoriasis by gender, age, and nation: (1) Global DALYs number, crude DALYs rate, and age-standardized DALYs rate from 1990 to 2017. (2) Global DALYs number, crude DALYs rate, and age-standardized DALYs rate between male and female from 1990 to 2017. (3) National DALYs number, crude DALYs rate, and age-standardized DALYs rate of each country from 1990 to 2017. (4) Gender- and age-specific crude DALYs rate in 2017.

### National Socioeconomic Status

The human development index (HDI) data is a comprehensive measure of health, education, and income, which was calculated based on four components: life expectancy at birth, mean years of schooling, expected years of education, and gross national income per capita. We used HDI as a national socioeconomic status indicator in this study. HDI data was collected from the United Nations Development Programme (UNDP; http://hdr.undp.org/en/data). We obtained the HDI data of each country. The HDI ranges from 0 to 1, with a higher value implying a higher socioeconomic development level. As the UNDP suggested, according to the value of HDI, countries were divided into four groups: very high human development (HDI ≥ 0.808), high (0.700 ≤ HDI < 0.808), medium (0.556 ≤ HDI < 0.700), and low (HDI < 0.556). We compared the psoriasis burden with each group.

### Measures of Health Inequality

The Gini coefficient and the concentration index were used to assess health inequality (9). Gini coefficient (also known as the Gini index or Gini ratio) is a measure of statistical dispersion based on the Lorenz curve intending to represent a nation's residents' income distribution. It is the most commonly used measure of inequality. The Gini coefficient ranges from 0 (perfect equality) to 1 (perfect inequality). The age-standardized DALYs rate caused by psoriasis for each country was used. The concentration index based on the concentration curve is a measure of the extent of socioeconomic-related inequality (10). The concentration index ranges from 0 (no inequality due to socioeconomic development) to 1 (complete inequality due to socioeconomic development). The concentration indexes were assessed by a national age-standardized DALYs rate caused by psoriasis and corresponding HDI, which calculated the correlation between psoriasis burden and socioeconomic development measured by HDI nationally. A positive/negative value represents that psoriasis burden is more concentrated in countries with high/low socioeconomic development levels. We computed the Gini coefficients and concentration indexes to explore the between-country health inequality trend from 1990 to 2017.

### Statistical Analysis

As the DALYs data tested non-normal distribution, the Kruskal–Wallis-test was performed to assess age-standardized DALYs rate differences across four HDI subgroups, followed by multiple comparisons using the Mann-Whitney U test with Bonferroni correction. The linear regression analysis was used to investigate the effect of HDI on age-standardized DALYs rate. Scatter plots demonstrated the relationship between psoriasis burden and HDI. The Gini coefficient and concentration index assessed health inequality. All statistical analyses, except multiple comparisons, were performed by STATA 15.0 SE (Stata Corp., College Station, TX, USA) and SPSS 23 (IBM Corp., Chicago, USA). A *p* < 0.05 was considered as statistically significant.

## Results

### Trends Over the Past 27 Years

**Figure 1:**
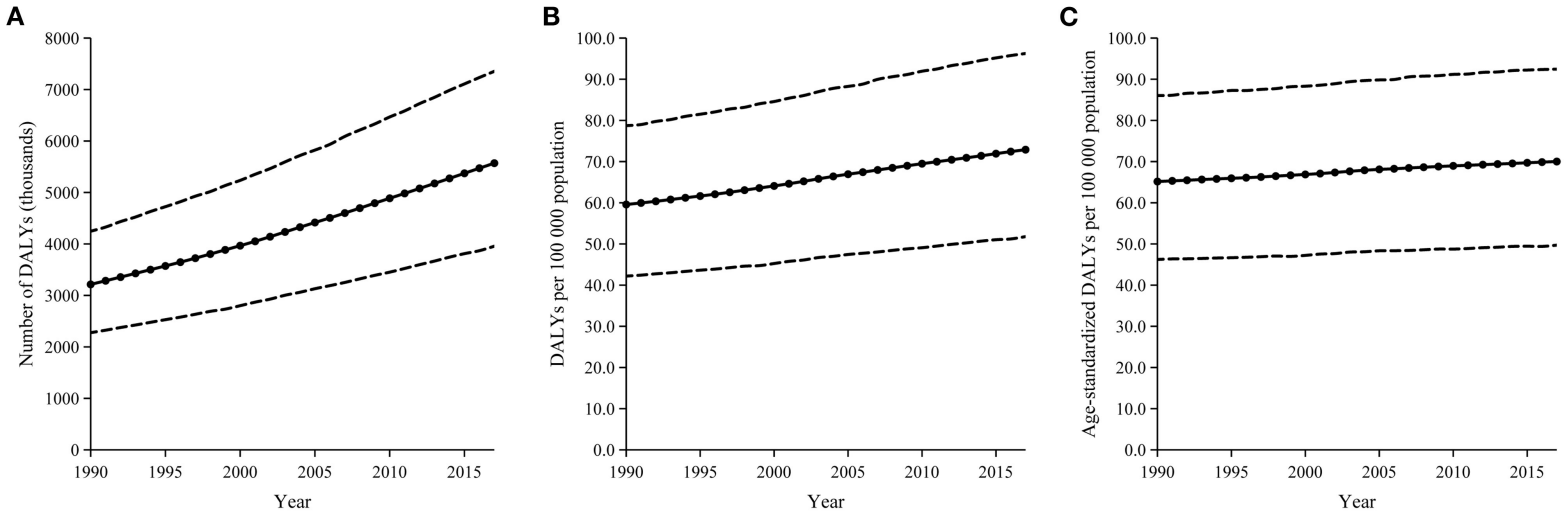
Trends in the global burden of cataract from 1990 to 2017, in terms of DALYs number **(A)**, crude DALYs rate **(B)**, and age-standardized DALYs rate **(C)**. The part between the dotted lines represents 95% uncertainty intervals.

As the global data showed: the DALYs number due to psoriasis increased by 73%, from 3,214,134 [95% uncertainty interval (UI): 2,276,373–4,247,062] to 5,569,471 (95% UI: 3,956,083–7,354,251) during the past 27 years (Figure 1A). The crude DALYs rate increased by 22% from 59.6 (95% UI: 42.2–78.7) in 1990 to 72.9 (95% UI: 51.8–96.3) in 2017 (Figure 1B). While the age-standardized DALYs rate showed a slight increase as 65.2 (95% UI: 46.2–86.0) in 1990 and 70.0 (95% UI: 49.7–92.5) in 2017 (Figure 1C).

### Global Burden Inequality by Age

**Figure 2:**
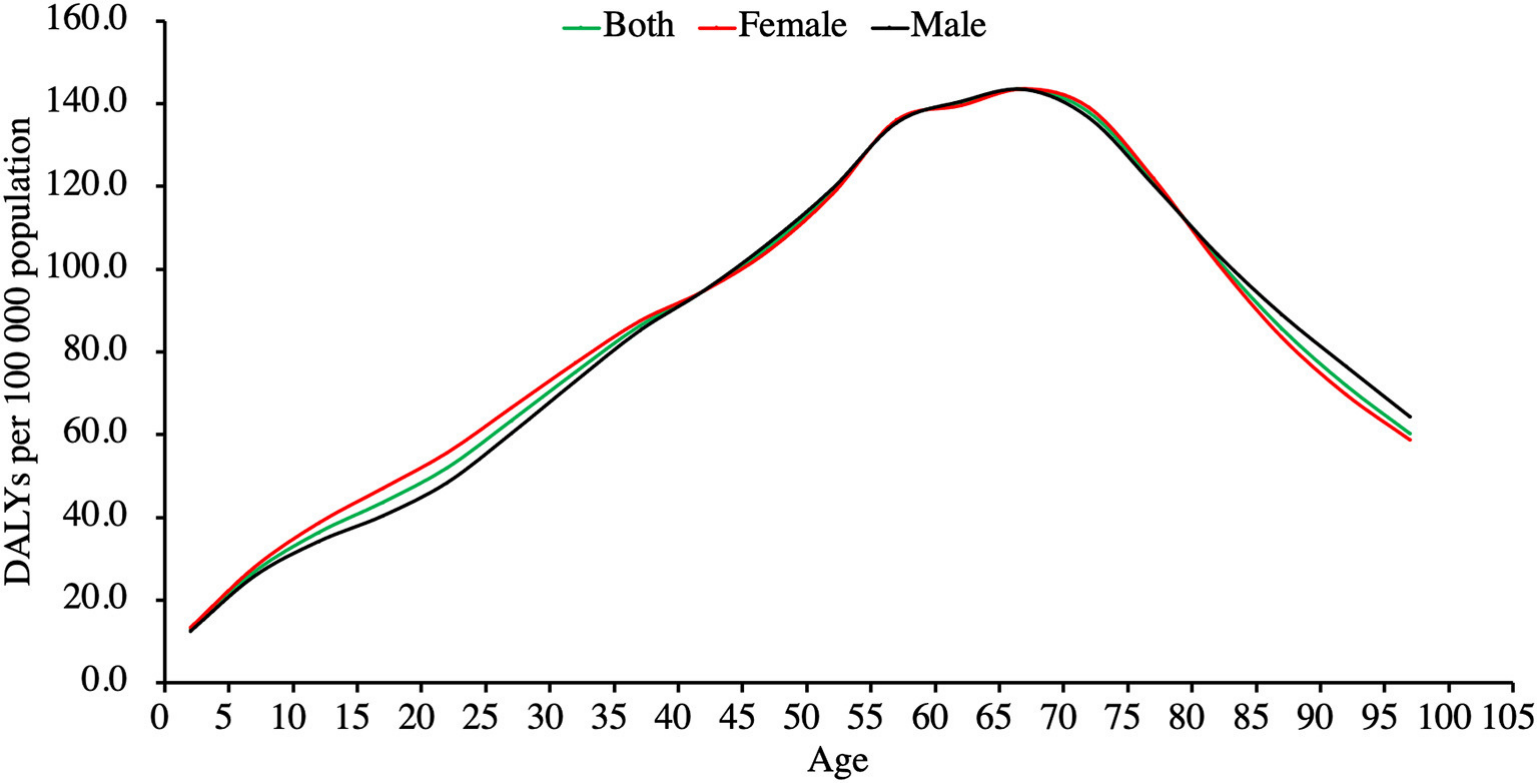
Global burden of psoriasis by age in 2017, in terms of crude DALYs rate.

In general, the global psoriasis burden had an increasing trend with age. After controlling for population size, the psoriasis burden peak was observed in the age range of 65–69 years, with 143.7 among females vs. 143.4 among males (Figure 2).

### Psoriasis Burden Inequality by Gender

**Figure 3:**
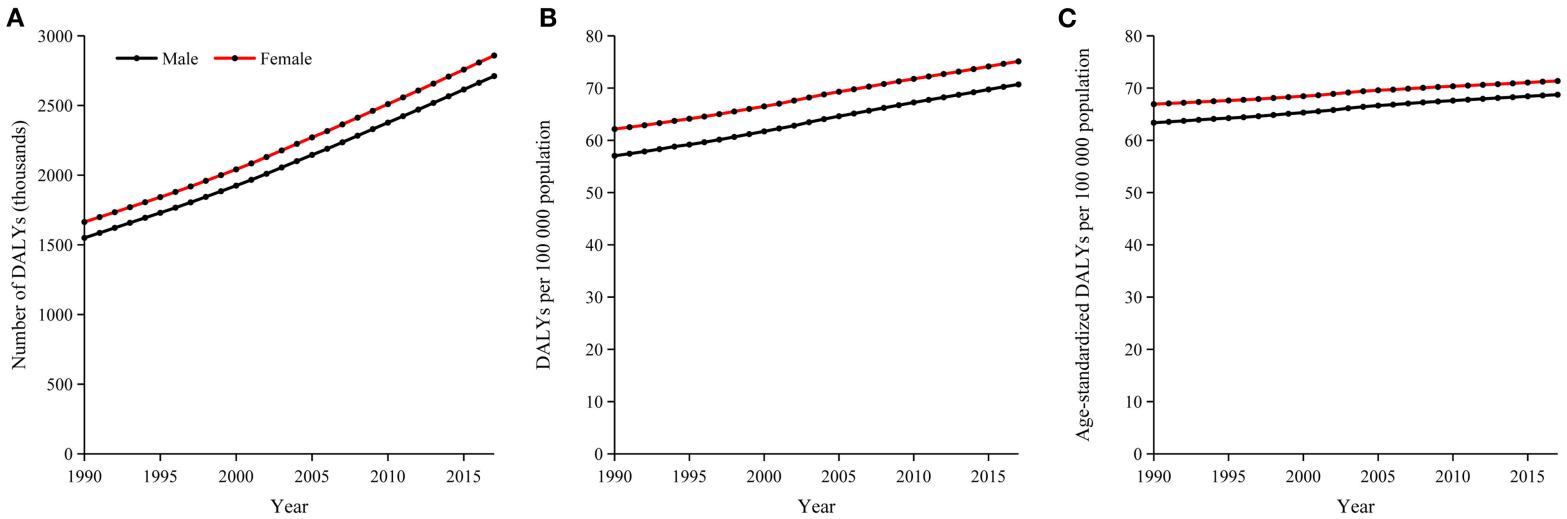
The persistence of gender inequality in global psoriasis burden from 1990 to 2017, in terms of DALYs number **(A)**, crude DALYs rate **(B)**, and age-standardized DALYs rate **(C)**.

**Figure 4:**
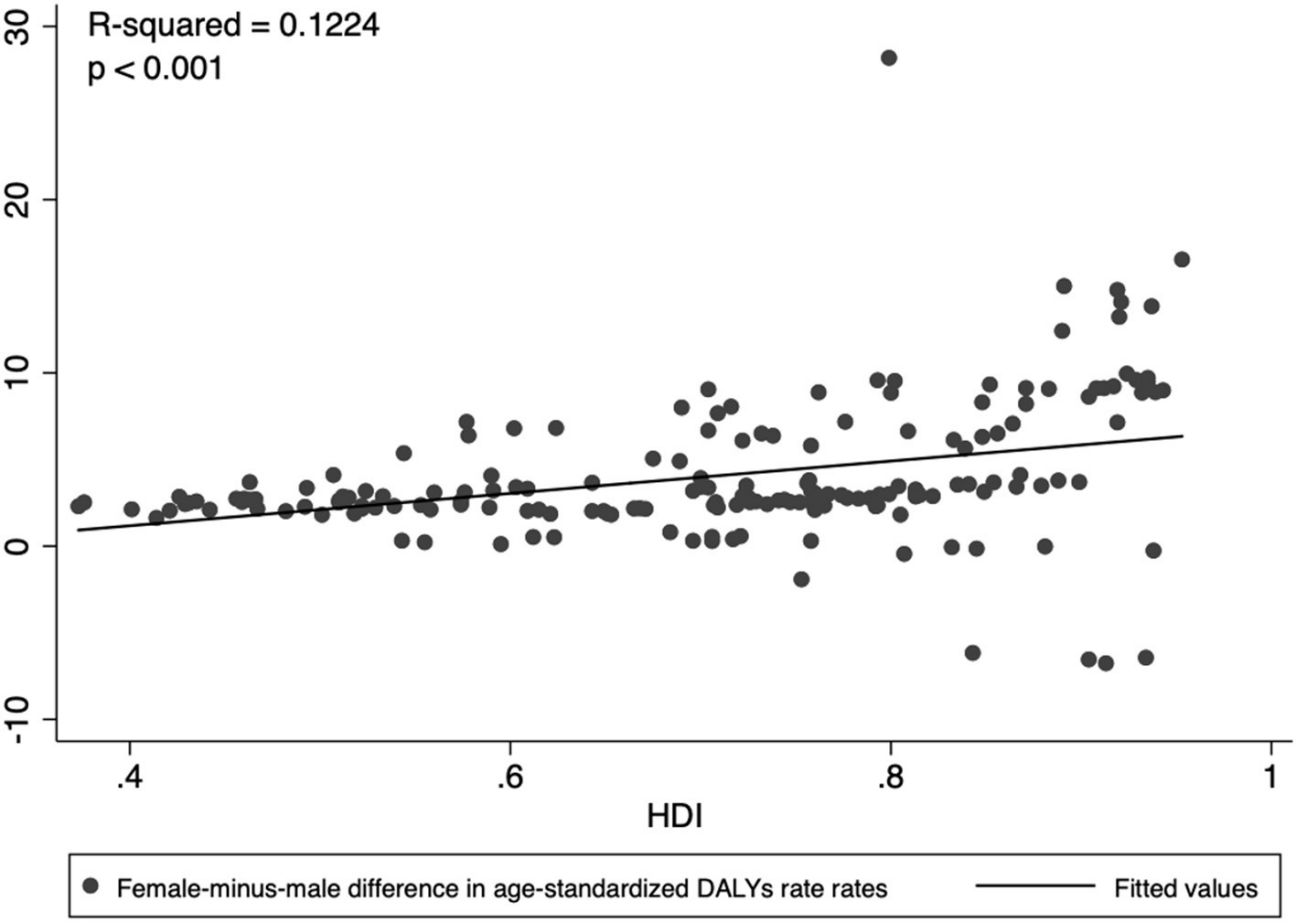
Female-minus-male difference in age-standardized DALYs rate showed a slightly positive inclination to the level of national socioeconomic development (R2 = 0.1224, *P* < 0.001).

Both males and females showed an increasing trend in the burden caused by psoriasis over the past 27 years. Gender inequality in the global burden of psoriasis was shown in DALYs number, crude DALYs rate, and age-standardized DALYs rate. The data revealed that gender inequality existed persistently over the past 27 years. The DALYs number of male/female was 1,550,155/1,663,979 in 1990 and increased to 2,710865/2 858 605 in 2017 (Figure 3A). The crude DALYs rate of male/female was 57.0/62.2 in 1990 and 70.7/75.1 in 2017 (Figure 3B). After controlling for both population size and age-structure, gender inequality was still shown in the age-standardized DALYs rate (Figure 3C). The age-standardized DALYs rate was 63.4 among males vs. 66.9 among females in 1990 and 68.7 vs. 71.4 in 2017. Analysis of linear regression demonstrated that female-minus-male difference in the age-standardized DALYs rate showed a slight incline in countries with a higher socioeconomic status (R2 = 0.1224, *P* < 0.001; Figure 4).

### Psoriasis Burden Inequality by Geographical Location

**Figure 5:**
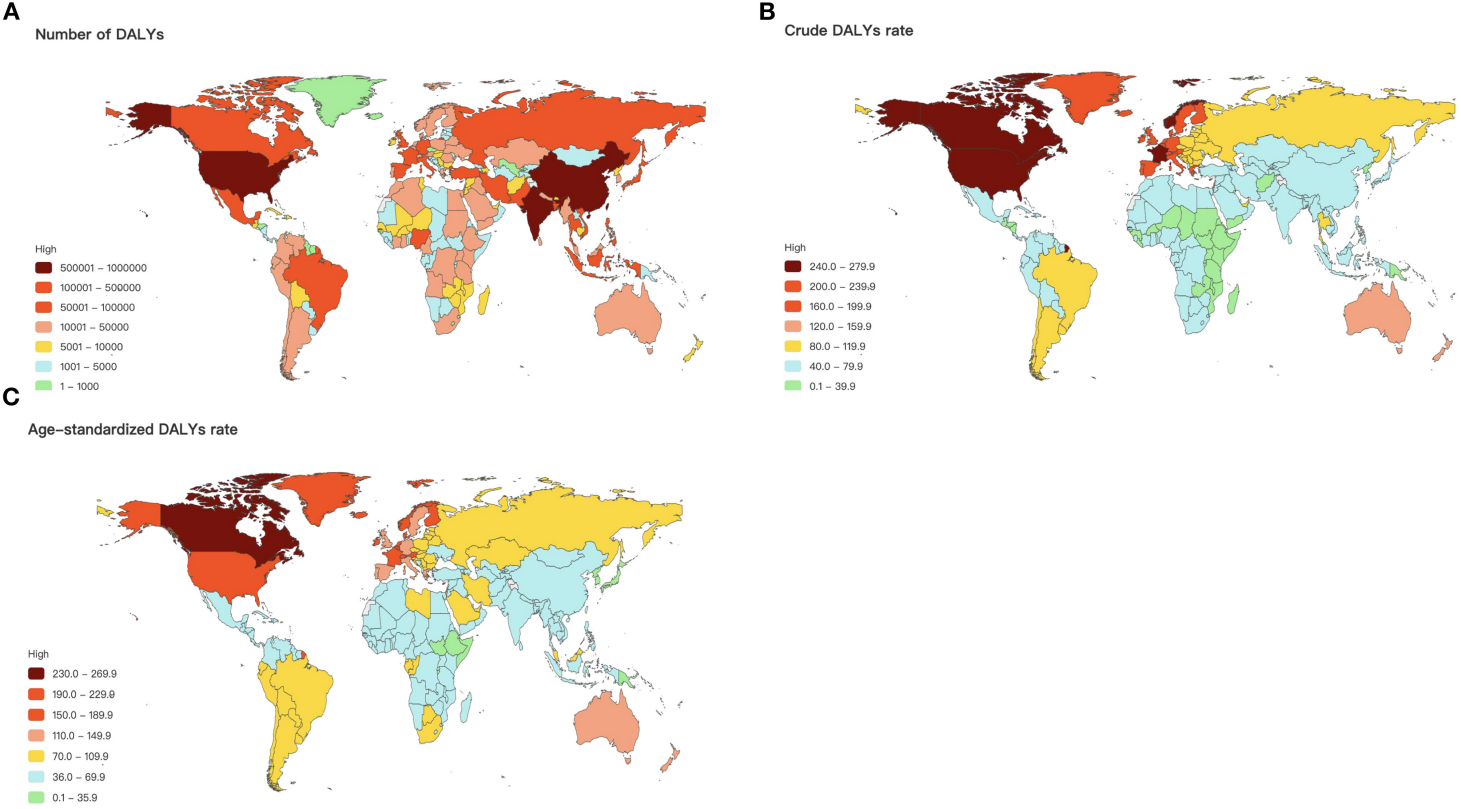
Global map of psoriasis burden in terms of DALYs number **(A)**, crude DALYs rate **(B)**, and age-standardized DALYs rate **(C)**.

Figure 5 shows the global distribution of psoriasis DALYs in 2017. The DALYs number was highest in the United States, China, India, Brazil, and France (Figure 5A). The crude DALYs rate was highest in Norway, Canada, the United States, followed by France and Greenland (Figure 5B). The age-standardized DALYs rate was similar to the crude DALYs rate, which was highest in Canada, the United States, Norway, France, and Greenland (Figure 5C). The health burden of psoriasis was substantially unequal in geography with a Gini coefficient of 0.27.

### Psoriasis Burden Inequality by Socioeconomic Status

**Figure 6:**
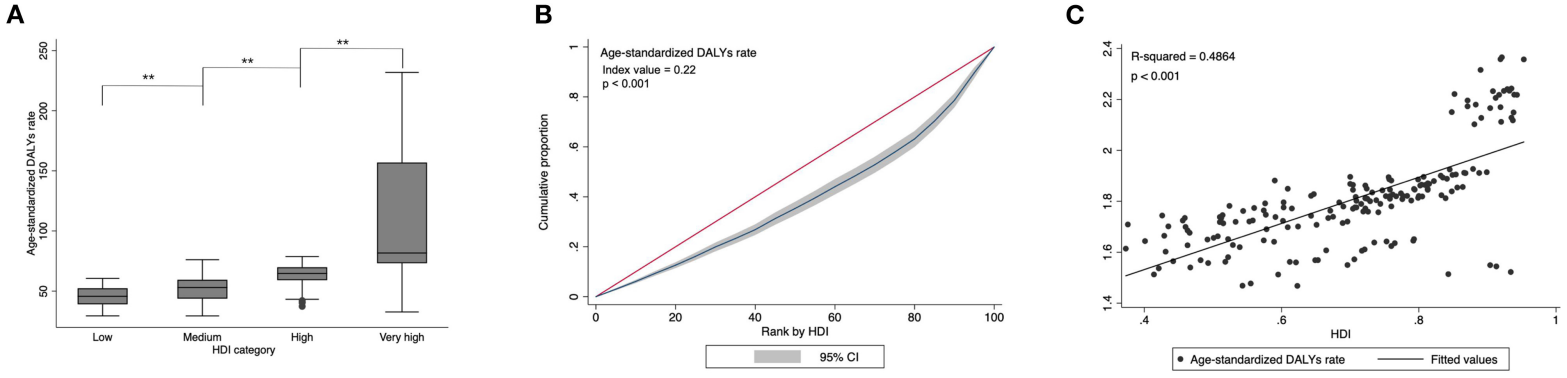
Psoriasis burden differed significantly across countries with different socioeconomic status levels. **P < 0.01 **(A)**. Concentration index indicated that socioeconomic associated inequality in psoriasis burden across countries with values of 0.22 **(B)**. Psoriasis burden was positively related to national socioeconomic development. The best-fitted line by linear regression analysis is presented **(C)**.

HDI data was available for 185 countries in 2017, including 59 very high, 52 high, 38 medium, and 36 low HDI countries according to the value of HDI. The Kruskal-Wallis test showed a significant difference in age-standardized DALYs rate across countries with different socioeconomic development levels (*P* < 0.01; Figure 6A). As shown in Figure 6B, the concentration index indicated socioeconomic associated inequality in psoriasis burden across countries with values of 0.22 in 2017. The concentration index's positive value suggested that psoriasis burden was more concentrated in countries with higher socioeconomic status. Analysis of linear regression demonstrated that age-standardized DALYs rate was positively related to HDI (R2 = 0.4864, *P* < 0.001; Figure 6C).

### Trends in Inequality in Psoriasis Burden

**Figure 7:**
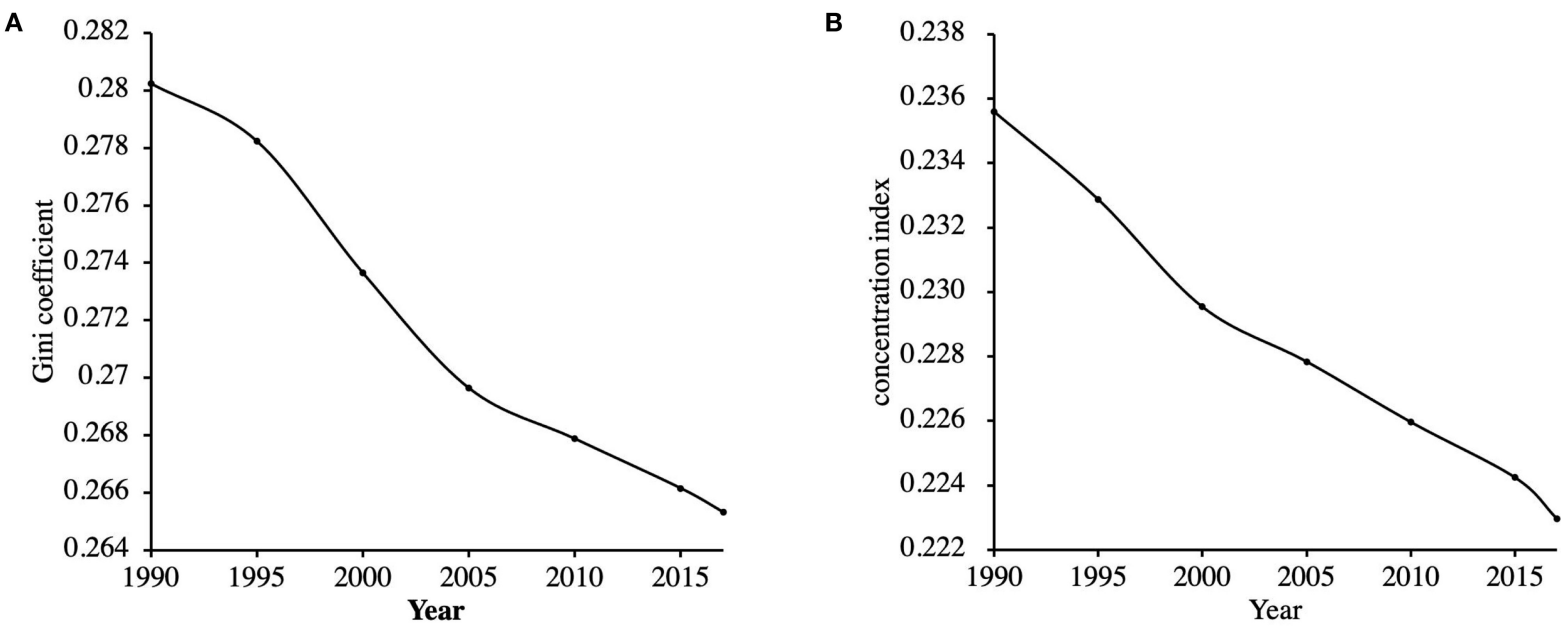
Trends in the Gini coefficients **(A)** and the concentration indexes **(B)** of psoriasis burden across countries from 1990 to 2017.

Although there was a slight increase in the global age-standardized DALYs rate during the past 27 years, between-nation inequality in the distribution of psoriasis burden kept declining during the same period. Gini coefficients of psoriasis burden decreased from 0.280 in 1990 to 0.265 in 2017 (Figure 7A). The concentration indexes indicated the same trend as 0.236 in the 1990s and 0.223 in 2017 (Figure 7B).

## Discussion

This study revealed the trends and variations of health burden due to psoriasis. The global burden of psoriasis has maintained a steady level without significant change since 1990 with inequality in age, gender, geography, and socioeconomic development.

Although the DALYs number and crude DALYs rate increased significantly, after controlling for both population size and age-structure, the result revealed that little had changed in the age-standardized DALYs rate during the past 27 years. Considering rapid population growth and an aging population, it is valid to use the age-standardized DALYs rate to evaluate the global psoriasis burden trend. With economic development and social progress, global healthcare improved in psoriasis, the global burden of psoriasis remained stable over a few decades. According to our study, it is reasonable to believe that with rapid socioeconomic development, growing sophistication in medical technology, and continuous improvement in environmental hygiene, psoriasis's global burden is well-controlled. Psoriasis can be chronic, and patients may easily relapse; therefore, psoriasis is usually associated with many other diseases, such as cardiovascular disease, obesity, diabetes mellitus II, dyslipidemia, non-alcoholic fatty liver disease, anxiety, depression, and inflammatory bowel disease (11, 12). Psoriasis can seriously influence the mental and physical health of patients. So how to reduce the psoriasis burden remains the focus of future work.

As to the impact of age, several articles had demonstrated prescription inequality due to the age-related drug efficiency or adverse events which may influence the type of psoriasis healthcare among different age ranges (13). The appearance of psoriasis forms and prevalence affected by age also play a role in the age inequality of psoriasis burden. Our study showed that patients in the age range of 65–69 usually showed economic inactivity, bearing a more significant psoriasis burden. Age highlights potential differences across the age groups.

Both males and females showed an increasing trend in burden caused by psoriasis over the past 27 years, with females bearing a more significant psoriasis burden than males. Gender inequality in psoriasis burden is also a focus which the whole society must pay attention to. According to our study, gender inequality in psoriasis burden persistently existed with little improvement during the past 27 years. The global health improvement of psoriasis does not mean less gender inequality. Moreover, linear regression analysis demonstrated that gender inequality showed a slight inclination to countries with higher socioeconomic status. Usually, females suffered a more significant physical and psychosocial impact of psoriasis on life quality than males (14–16). Researches showed that even though the clinical manifestations were lighter than in males, females bore more significant psychological distress and a higher probability of anxiety or depression (14). Among psoriasis patients, females may be more prone to perceive stress and may be more likely to perceive a more significant mental impact. In all regions of the world and at all time periods, the longevity pattern showed that females live longer than males, and therefore females suffered and were affected by poor health for longer (8).

Furthermore, besides the mental impact, psoriasis patients often have skin discomfort and joint pain, which is detrimental to work productivity and has an associated cost burden (17). Patients reported that psoriasis localized to their hands or feet caused work limitations and caused them to quit their job (18). Patients had limited expectations of career progression. Psoriasis reduced employment status prospects and earning potential and raised the cost due to clinical assessment or treatment. Females are more sensitive to the physical and mental symptoms caused by psoriasis; thus, they usually suffer a more severe impact on their career and higher health care cost than males.

As our study showed, although the socioeconomic disparity of psoriasis burden whittled during the past 27 years, inequality by geographical location and socioeconomic status remains a significant concern for reducing psoriasis' global burden. Previous literature has reported that psoriasis's global burden varied little in geography in 2010 when comparing the psoriasis-associated DALYs rate in 21 geographical regions (1). To provide a more accurate and detailed assessment of the geographic differences, we used each countries' age-standardized DALYs rate for national comparison. Unlike the theoretical distribution of equivalence, the Lorenz curve indicated a geographical distribution of psoriasis burden with an associated Gini coefficient of 0.27 in 2017. The concentration indexes showed socioeconomic associated inequality in psoriasis burden across countries with values of 0.22 in 2017. To further probe the relationship between psoriasis burden and national socioeconomic status, we had also tested for a linear relationship between psoriasis burden and national socioeconomic status. The result showed that a higher psoriasis burden was concentrated in countries with higher levels of socioeconomic development.

This pattern is concerning, given the lack of substantial improvements in age-standardized rates with social-economic development. It is probably due to population growth and aging, which is more evident in socioeconomically developed areas. Additionally, environmental factors and diet structures may also contribute to the phenomenon. Previous research reported that high cholesterol, poor diet, high fasting plasma glucose, obesity, and low physical activity (19) related to psoriasis prevalence to some degree prevail in many high-income countries.

This study provides global population-based estimates of psoriasis burden using the most reliable epidemiologic data currently available. Our study results reveal trends in the inequality in age, gender, geography, and socioeconomic development of psoriasis burden, which is worth attention. The finding may increase the awareness of psoriasis status, drive continued national and international dialogue, and target efforts toward improving psoriasis services. However, the limitations of this study should also be stressed. First, the psychosocial impact caused by psoriasis (20) is often neglected, and the harm of psoriasis associated diseases is not included in the evaluation of psoriasis burden by GBD (1). Second, the estimation by GBD is so subjected to the number and quality of the included population that the accuracy of this data needs to be confirmed. Undiagnosed and untreated psoriasis is also a factor that affects the results. When considering all the above factors, the health burden of psoriasis may be underestimated. Additionally, the article was completed before the GBD database was updated to 2019. As the database was updated with the dataset change, each year's original data changed, so we did not add the modified data in our research. Furthermore, we are now comparing the data before and after the GBD database update to analyze the possible factors that influence the psoriasis burden such as comorbidities, to find methods to estimate disease burden more precisely and efficiently in the future.

Despite these constraints, this study reveals global health progress in psoriasis, together with inequalities in the past few decades. Although the inequality of psoriasis burden has shown some improvement during the past 27 years, the disparity still exists in age, gender, geographical location, and socioeconomic status. Regarding psoriasis treatment, psoriatic treatments at present range from topical treatments, systemic treatments (including methotrexate, cyclosporine, and biologic agents) to phototherapy. We emphasize individualized treatment but lack large-scale data analysis on a global scale. The study of epidemiological variations in psoriasis' global burden provides valid theoretical support in individualized treatment planning.

This study highlights psoriasis's global importance and is essential in policy planning for psoriasis service on a global scale.

## Data Availability Statement

Publicly available datasets were analyzed in this study. This data can be found here: http://ghdx.healthdata.org/gbd-results-tool.

## Author Contributions

CP, WC, and JL are responsible for the guarantor of integrity of the entire study, study concepts and design, definition of intellectual content, literature research, clinical studies, data analysis and collection, statistical analysis, manuscript preparation and editing, and review. XL, NY, XY, and YD are responsible for the study design, literature research, data acquisition, and manuscript preparation. All authors approved this manuscript.

## Conflict of Interest

The authors declare that the research was conducted in the absence of any commercial or financial relationships that could be construed as a potential conflict of interest.
